# Early Response Assessment in Advanced Stage Melanoma Treated with Combination Ipilimumab/Nivolumab

**DOI:** 10.3389/fimmu.2022.860421

**Published:** 2022-07-06

**Authors:** Vincent T. Ma, Alahendra A. Chamila Perera, Yilun Sun, Merna Sitto, Jessica J. Waninger, Govind Warrier, Michael D. Green, Leslie A. Fecher, Christopher D. Lao

**Affiliations:** ^1^ Department of Internal Medicine, Division of Hematology, Medical Oncology, and Palliative Care, University of Wisconsin, Madison, WI, United States; ^2^ Department of Internal Medicine, Division of Hematology and Oncology, University of Michigan, Ann Arbor, MI, United States; ^3^ Department of Biostatistics, University of Michigan, Ann Arbor, MI, United States; ^4^ Department of Radiation Oncology, University of Michigan, Ann Arbor, MI, United States; ^5^ Department of Medical Education, University of Michigan, Ann Arbor, MI, United States; ^6^ Department of Internal Medicine, University of Michigan, Ann Arbor, MI, United States; ^7^ Department of Radiation Oncology, Veteran Affairs Ann Arbor Hospital System, Ann Arbor, MI, United States

**Keywords:** melanoma, biomarker, radiological assessment, combination immune checkpoint therapy, ipilimumab and nivolumab, early response assessment, immune related adverse effects

## Abstract

**Background:**

Standard combination ipilimumab/nivolumab (I/N) is given as 4 induction doses for advanced stage melanoma followed by nivolumab single-agent maintenance therapy. While many patients receive less than 4 doses due to immune-related toxicities, it is unclear if fewer doses of I/N may still provide long term clinical benefit. Our aim is to determine if response assessment after 1 or 2 doses of I/N can predict long-term survival and assess if fewer doses of I/N can lead to similar survival outcomes.

**Methods:**

We performed a retrospective analysis on a cohort of patients with advanced melanoma who w0ere treated with standard I/N. Cox regression of progression-free survival (PFS) and overall survival (OS) models were performed to assess the relationship between response after 1 or 2 doses of I/N and risk of progression and/or death. Clinical benefit response (CBR) was assessed, defined as SD (stable disease) + PR (partial response) + CR (complete response) by imaging. Among patients who achieved a CBR after 1 or 2 doses of I/N, a multivariable Cox regression of survival was used to compare 1 or 2 vs 3 or 4 doses of I/N adjusted by known prognostic variables in advanced melanoma.

**Results:**

199 patients were evaluated. Patients with CBR after 1 dose of I/N had improved PFS (HR: 0.16, 95% CI 0.08-0.33; p<0.001) and OS (HR: 0.12, 0.05-0.32; p<0.001) compared to progressive disease (PD). Patients with CBR (vs PD) after 2 doses of I/N also had improved PFS (HR: 0.09, 0.05-0.16; p<0.001) and OS (HR: 0.07, 0.03-0.14; p<0.001). There was no survival risk difference comparing 1 or 2 vs 3 or 4 doses of I/N for PFS (HR: 0.95, 0.37-2.48; p=0.921) and OS (HR: 1.04, 0.22-4.78; p=0.965).

**Conclusions:**

Early interval imaging with response during induction with I/N may be predictive of long-term survival in advanced stage melanoma. CBR after 1 or 2 doses of I/N is associated with favorable survival outcomes, even in the setting of fewer I/N doses received. Further studies are warranted to evaluate if electively administering fewer combination I/N doses despite tolerance in select patients may balance the benefits of therapy while decreasing toxicities.

## Introduction

Immune checkpoint inhibitors (ICIs) are standard of care for treatment of advanced melanoma. Combined inhibition of cytotoxic T lymphocyte antigen-4 (CTLA-4) and programmed death 1 (PD-1) with ipilimumab and nivolumab has been shown to be highly effective for treatment of unresectable stage III or IV melanoma (1, 2). Results from the CheckMate-067 trial found that previously untreated, advanced melanoma patients had statistically improved objective response rate (ORR), progression-free survival (PFS), and overall survival (OS) with combination ipilimumab/nivolumab (I/N) versus ipilimumab monotherapy (3, 4). Although the trial was not designed to compare the nivolumab-containing cohorts, descriptive subgroup analyses showed superior PFS and OS with I/N compared to nivolumab alone (3). Recently published data found more than half the patients receiving I/N were alive at 5-year follow up (2). Based on these results, I/N is frequently used as first-line treatment of advanced stage melanoma.

Despite its apparent greater efficacy, combination therapy comes at the expense of considerably higher immune-related adverse events (irAEs) than the respective single-agent counterparts. Rates of grade 3-4 treatment-related toxicities in I/N, nivolumab, and ipilimumab were 59%, 22%, and 28%, respectively (4). Similar rates of toxicities were also noted in the CheckMate-218 North American cohort (5). Standard combination I/N for advanced melanoma is given as four induction doses with ipilimumab and nivolumab once every 3 weeks, followed by nivolumab monotherapy (2, 6). However, a substantial number of patients are unable to receive all 4 doses due to irAEs. In the trials, 41-61% of patients received fewer than 4 induction doses (4, 5). At present, differential clinical outcomes in patients receiving fewer combination I/N doses received, for reasons other than toxicity, remains uncertain.

In the CheckMate-067 and CheckMate-069 trials, timing of response assessment by imaging was conducted post-induction with I/N (2, 7). Several studies have investigated the utility of various radiographic imaging techniques in early detection of response to chemotherapeutic, targeted, and immunotherapeutic agents in a variety of tumor types (8–10). The utility of interval imaging while receiving dual-agent ICI therapy is unclear. One possible limitation is the phenomenon of tumor flare of pseudoprogression, where disease response occurs after an initial increase in tumor burden during treatment with ICI, which can be misinterpreted as progressive disease depending on the timing of imaging assessments. Currently, there are no clear guidelines on optimal timing or appropriateness of early response assessment. As such, response-adapted therapy remains a relatively unexplored treatment strategy in advanced melanoma management or any other malignancies treated with combination checkpoint blockade.

In our study, we examine if early response assessment after 1 or 2 doses of I/N is predictive of long-term survival. We also attempt to determine if receipt of fewer I/N doses is associated with a similar survival outcome as the standard 4 induction doses.

## Methods

### Study Population

We identified 548 patients with histologically proven unresectable stage III or IV melanoma following American Joint Committee on Cancer (AJCC) 8th edition criteria who were treated with ICI from February 2012 to December 2020 at the University of Michigan (11). Uveal melanoma patients were excluded. Among the cohort, 199 patients were treated with standard I/N and were evaluated in our retrospective study. The combination I/N dosing was ipilimumab 3 mg/kg and nivolumab 1 mg/kg every 3 weeks up to 4 doses followed by nivolumab therapy at 3 mg/kg every 2 weeks or 240 mg every 2 weeks or 480 mg every 4 weeks. All patients received at least 1 dose of induction I/N. Patients with insufficient clinical data or follow up (less than 30 days) from initiation of therapy were excluded.

Patients and data were collected *via* electronic medical record system and a pharmacy database hosted by the University of Michigan. The cohort included patients who were treatment naïve or previously treated with other systemic agents, including in the adjuvant setting, before receiving combination I/N. Prior systemic agents included: high-dose interferon, interleukin 2 (IL-2), ipilimumab monotherapy, pembrolizumab monotherapy, nivolumab monotherapy, vemurafenib, or dabrafenib +/- trametinib.

### Study Design

Baseline patient characteristics and demographics included age, gender, BRAF mutation status, primary melanoma type, and prior treatment status (**Table 1**). Prognostic factors including AJCC stage, pre-treatment serum lactate dehydrogenase level (LDH), presence of brain metastasis, and presence of liver metastasis were documented before initiation of I/N. Efficacy end-points of treatment were objective response, progression-free survival (PFS), and overall survival (OS).

**Table 1:** Patient characteristics.

		All	Total number of I/N doses received				P-value*
			1	2	3	4	
**n (%)**		199 (100%)	28 (100%)	68 (100%)	44 (100%)	59 (100%)
**Stage** ^†^							0.9340
	Stage III, unresectable	11 (6%)	2 (7%)	4 (6%)	2 (5%)	3 (5%)	
	Stage IV (M1a)	10 (5%)	3 (11%)	3 (4%)	3 (7%)	1 (2%)	
	Stage IV (M1b)	38 (19%)	6 (21%)	12 (18%)	9 (20%)	11 (19%)	
	Stage IV (M1c)	92 (46%)	10 (36%)	34 (50%)	18 (41%)	30 (51%)	
	Stage IV (M1d)	48 (24%)	7 (25%)	15 (22%)	12 (27%)	14 (24%)	
**Systemic Treatment Naïve**							0.5796
	Yes	162 (81%)	22 (79%)	54 (79%)	39 (89%)	47 (80%)	
	No	37 (19%)	6 (21%)	14 (21%)	5 (11%)	12 (20%)	
**Age**							0.8038
	<65	150 (75%)	22 (79%)	53 (78%)	31 (70%)	44 (75%)	
	≥65	49 (25%)	6 (21%)	15 (22%)	13 (30%)	15 (25%)	
**Gender**							0.6653
	Male	129 (65%)	10 (36%)	51 (75%)	21 (48%)	47 (80%)	
	Female	70 (35%)	18 (64%)	17 (25%)	23 (52%)	12 (20%)	
**BRAF mutation status**							0.1423
	V600 mutant	87 (44%)	14 (50%)	32 (47%)	20 (45%)	21 (36%)	
	Wildtype	108 (54%)	14 (50%)	36 (53%)	21 (48%)	37 (63%)	
	Unknown	4 (2%)	0	0	3 (7%)	1 (2%)	
**Primary melanoma type**							0.9037
	Cutaneous	158 (79%)	22 (79%)	55 (81%)	36 (82%)	44 (75%)	
	Mucosal	19 (10%)	2 (7%)	5 (7%)	5 (11%)	6 (10%)	
	Unknown	22 (11%)	4 (14%)	8 (12%)	3 (7%)	8 (14%)	
**Pre-treatment LDH level**‡							0.5965
	Normal	120 (60%)	17 (61%)	41 (60%)	29 (66%)	33 (56%)	
	> Upper limit of normal	66 (33%)	8 (29%)	25 (37%)	13 (30%)	20 (34%)	
	Unknown	13 (7%)	3 (11%)	2 (3%)	2 (5%)	6 (10%)	
**Liver Metastases**							0.5666
	Yes	80 (40%)	9 (32%)	28 (41%)	18 (41%)	22 (37%)	
	No	119 (60%)	19 (68%)	40 (59%)	26 (59%)	37 (63%)	
**Brain Metastases**							0.9374
	Yes	48 (24%)	7 (25%)	15 (22%)	12 (27%)	14 (24%)	
	No	151 (76%)	21 (75%)	53 (78%)	32 (73%)	45 (76%)	

I/N, ipilimumab/nivolumab; LDH, lactate dehydrogenase.

^†^Per AJCC 8th edition.

^‡^Normal LDH level is <240 IU/L.

^*^Chi-squared test.

At the University of Michigan, disease reassessment with imaging (CT or MRI) is frequently performed following the 1st and/or 2nd dose of I/N to guide treatment decisions (i.e. if moderate toxicities are present but response is seen, then switching to nivolumab monotherapy is considered). For the purposes of this study, we applied the revised RECIST guideline (version 1.1) (12), with the noted exception of following the largest target lesions in retrospect, with assessments of complete response (CR), partial response (PR), stable disease (SD), or progressive disease (PD). Time to initial assessment was measured from I/N treatment start date to date of initial response assessment (1st or 2nd dose, whichever occurred first). Patients defined as having a response assessment after 1 dose of I/N temporally occurred between the 1st and 2nd dose of I/N, but if a 2nd dose was never received, then the response was assessed within 30 days after the 1st dose of I/N. For patients who had a response assessment after 2 doses of I/N, this occurred between the 2nd and 3rd dose of I/N, but if a 3rd dose was never received, then the response was assessed within 30 days after the 2nd dose of I/N.

PFS was defined as the time from the date of I/N initiation to clinical progression on imaging based on the iRECIST criteria (13), or date of death, whichever occurred first. OS was determined based on electronic health record documentation. Patients who were alive at the time of the analysis were censored at last known follow up. Response and progression date evaluations were documented and verified by two independent reviewers. Discordant results were resolved by a third reviewer.

### Statistical Methods

PFS and OS were assessed using Kaplan-Meier methods. A univariable Cox regression of PFS and OS models was performed to assess the effect of objective response after 1 and 2 doses of I/N. A distinct variable for disease response was defined as clinical benefit response (CBR), which encompasses SD (stable disease) + PR (partial response) + CR (complete response) by imaging (CT or MRI) per RECIST v1.1.

Univariate and multivariable Cox regression of survival was used to compare 1 or 2 vs 3 or 4 doses of I/N and the following baseline and prognostic variables: age, gender, pre-treatment LDH level, BRAF mutation status, primary melanoma type, time to initial assessment, presence of brain metastasis, and presence of liver metastasis. A repeat univariate and multivariable Cox regression assessment was performed among the cohort of patients who achieved a CBR after 1 or 2 doses of I/N. In our supplementary analysis, we used Spearman’s rank coefficient to correlate response assessment (*via* RECIST v1.1) after 1 and 2 dose of I/N with future best response (*via* iRECIST).

## Results

199 melanoma patients treated with I/N were evaluated in our study (**Table 1**). Median follow up period was 28.8 months (interquartile range [IQR]: 13.7-43.3 months). The median age was 56 years old (IQR: 46-64 years old). 59 (30%) patients received all 4 induction doses of I/N. Of the remaining patients, 44 (22%) received 3 doses, 68 (34%) received 2 doses, and 28 (14%) received 1 dose. 162 (81%) patients were naïve to any prior systemic therapy. 158 (79%) patients had primary cutaneous melanoma; the remainder were either mucosal or unknown. In our cohort, 80 (40%) patients had liver metastases and 48 (24%) patients had brain metastases prior to starting I/N.

See **Table 2** for patient treatment response and toxicity. Among the patients who received 1, 2, 3, and 4 doses of I/N; 54%, 56%, 61%, and 85% went on to receive maintenance nivolumab therapy, respectively. Of the 140 patients who received fewer than 4 induction I/N doses, 80 (57%) patients continued on to maintenance nivolumab monotherapy and 60 (43%) patients did not. IrAEs as the cause for receiving fewer than 4 doses of I/N occurred in 75%, 75%, and 80% of patients who received 1, 2, and 3 doses, respectively. The grade 3+ immune-related toxicity rate was 48% for all patients. PD or death was the reason for fewer than 4 doses for the remainder of patients. In our cohort, 49% (97/199) had imaging assessments after 1 dose of I/N and 68% (135/199) had imaging assessments after 2 doses of I/N. The vast majority of patients had early radiographic response assessment using computed tomography (CT) scans (128/135), whereas a small fraction used MRI (5/135) and PET/CT (2/135) imaging.

**Table 2:** Patient treatment response and toxicity.

		All	Total number of I/N doses received			
			1	2	3	4
**n (%)**		199 (100%)	28 (100%)	68 (100%)	44 (100%)	59 (100%)
**Received maintenance nivolumab**						
	Yes	130 (65%)	15 (54%)	38 (56%)	27 (61%)	50 (85%)
	No	69 (35%)	13 (46%)	30 (44%)	17 (39%)	9 (15%)
Number of maintenance nivolumab doses*						
	Minimum	0	0	0	0	0
	25th percentile	0	0	0	0	1
	Median	4	4	2	1	12
	75th percentile	16	11	13	13	21
	Maximum	54	44	51	42	54
**Reason for <4 doses of I/N**						
	Immune-related adverse event	–	21 (75%)	51 (75%)	35 (80%)	–
	Progressive disease or death	–	7 (25%)	17 (25%)	9 (20%)	–
**Grade toxicity**†						
	Any	165 (83%)	24 (86%)	58 (85%)	39 (89%)	44 (75%)
	Grade 3-5	79 (48%)	14 (50%)	22 (32%)	22 (50%)	16 (27%)
**Time to Initial Assessment (months)**						
	Median (IQR)	0.76 (0.59-1.35)	0.69 (0.59-0.95)	0.89 (0.62-1.24)	0.76 (0.65-1.40)	0.72 (0.59-1.68)
	Mean	1.08	0.80	1.03	1.18	1.21
**Early Response** ^‡^						
	CBR after 1 dose of I/N	52 (26%)	13 (46%)	15 (22%)	11 (25%)	13 (22%)
	PD after 1 dose of I/N	45 (23%)	10 (36%)	15 (22%)	6 (14%)	14 (24%)
	Response not evaluated after 1 dose of I/N	102 (51%)	5 (18%)	36 (56%)	27 (61%)	32 (54%)
	CBR after 2 doses of I/N	92 (46%)	–	42 (62%)	23 (52%)	27 (46%)
	PD after 2 doses of I/N	43 (22%)	–	23 (34%)	7 (16%)	13 (22%)
	Response not evaluated after 2 doses of I/N	36 (18%)	–	3 (4%)	14 (32%)	19 (32%)
**Best Response^§^**						
	CR	70 (35%)	10 (36%)	20 (29%)	18 (41%)	22 (37%)
	PR	74 (37%)	5 (18%)	26 (38%)	18 (41%)	25 (42%)
	SD	14 (7%)	4 (14%)	3 (4%)	1 (2%)	6 (10%)
	PD	41 (21%)	9 (32%)	19 (28%)	7 (16%)	6 (10%)

I/N, ipilimumab/nivolumab; CBR, clinical benefit response; PD, progressive disease; IQR, interquartile range.

^*^Nivolumab 240 mg = 1 dose; nivolumab 480 mg = 2 doses.

^†^Common Terminology Criteria for Adverse Events (CTCAE) version 5.0.

^‡^Per RECIST v1.1 criteria.

^§^Per iRECIST criteria.

**Figure 1:**
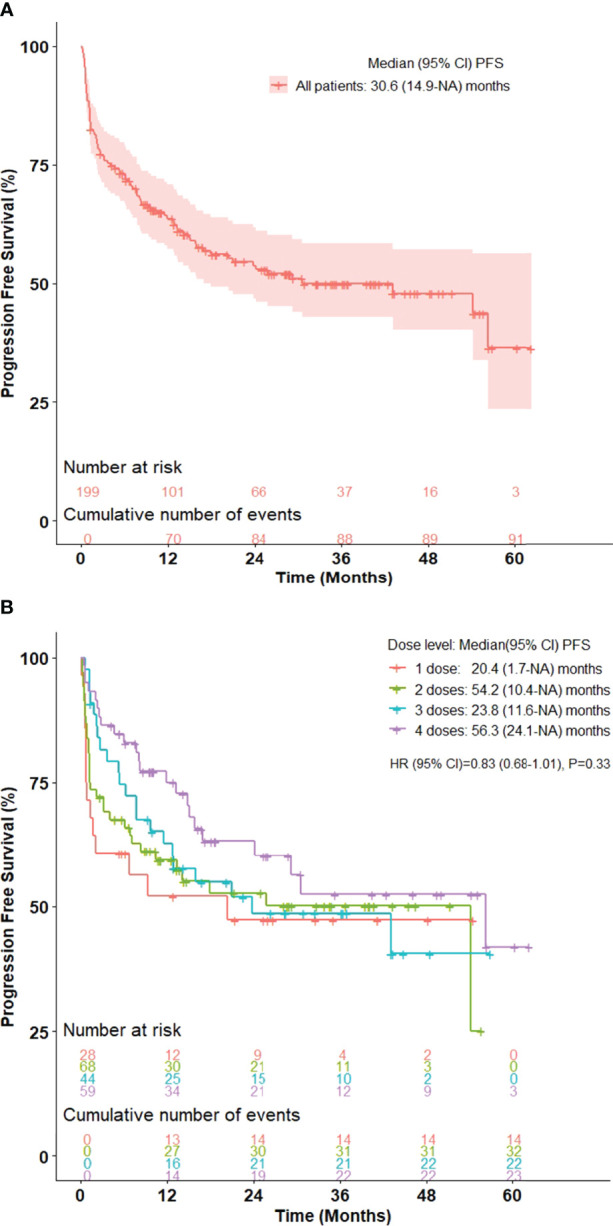
Kaplan-Meier curve for progression-free survival in advanced stage melanoma patients treated with ipilimumab/nivolumab in **(A)** all patients and **(B)** stratified by number of ipilimumab/nivolumab doses received. n = 199.

**Figure 2:**
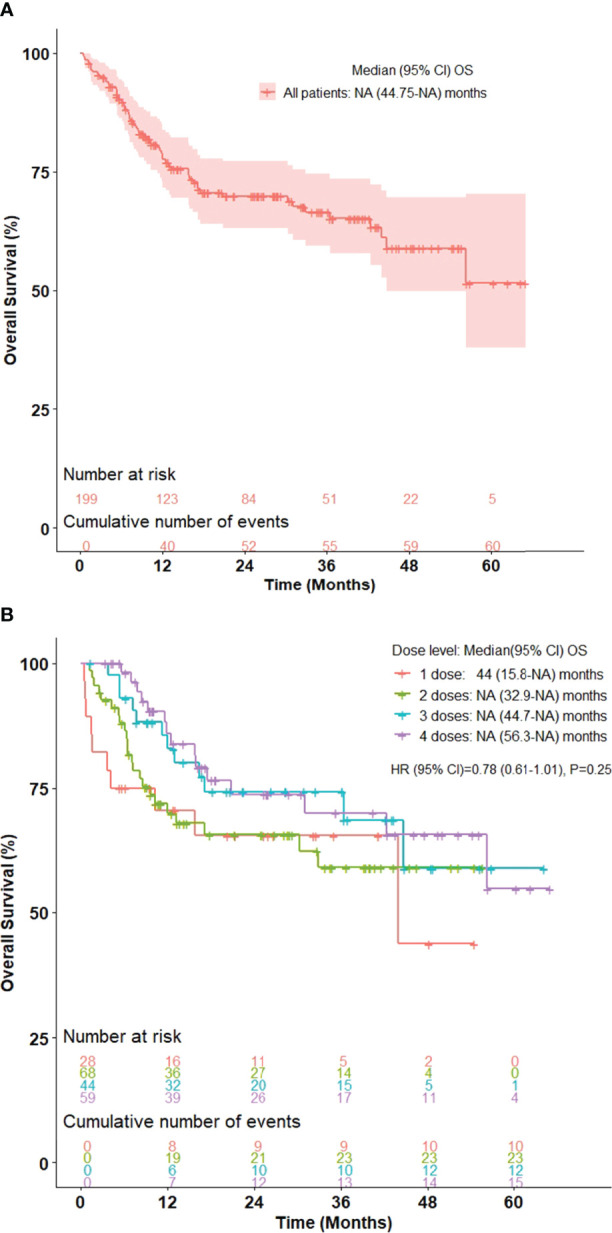
Kaplan-Meier curve for overall survival in advanced stage melanoma patients treated with ipilimumab/nivolumab in **(A)** all patients and **(B)** stratified by number of ipilimumab/nivolumab doses received. n = 199.

Among all 199 patients, the 36-month and 60-month PFS was 50.0% (95% CI, 42.7-58.5) and 36.3% (95% CI, 23.3-56.4), respectively (**Figure 1A**). The 36-month PFS for patients receiving 1, 2, 3, and 4 doses were 47.3%, 50.1%, 48.6%, and 52.4%, respectively (**Figure 1B**). The 36-month and 60-month OS for the entire cohort was 66.5% (95% CI, 59.3-74.6) and 51.6% (95% CI, 37.8-70.3), respectively (**Figure 2A**). The 36-month OS for patients receiving 1, 2, 3, and 4 doses were 65.5%, 59.0%, 74.3%, and 70.1%, respectively (**Figure 2B**).

Among all evaluable patients (n=118) with complete baseline characteristic and prognostic information, univariate Cox regression (**Supplementary Table 1**) and multivariable Cox regression analyses (**Table 3**) were performed. The multivariable Cox regression analysis demonstrated that PD (vs CBR) as initial response after 1 or 2 doses of I/N [HR: 7.62, 95% CI, 3.71-15.66; p<0.0001], treatment with 1 or 2 doses (vs 3 or 4 doses) of I/N [HR: 2.75, 95% CI, 1.47-5.13; p=0.002], presence of brain metastases [HR: 2.01, 95% CI, 1.09-3.72; p=0.025], and presence of liver metastases [HR: 2.01, 95% CI, 1.04-3.88; p=0.038] were associated with inferior PFS.

**Table 3:** Multivariable Cox regression of progression-free survival and overall survival on the number of I/N (ipilimumab/nivolumab) doses and prognostic variables among patients who had response assessment after 1 or 2 doses of I/N. n = 118.

	Progression-Free Survival		Overall Survival	
Variable	Hazard Ratio (95% CI)	p-value	Hazard Ratio (95% CI)	p-value
**PD vs. CBR initial assessment**†	7.62 (3.71-15.66)	<0.0001*	8.04 (3.31-19.53)	<0.0001*
**I/N doses (1 or 2 vs. 3 or 4)**	2.75 (1.47-5.13)	0.002*	2.09 (1.04-4.19)	0.038*
**Time to initial assessment**	0.62 (0.30-1.28)	0.199	1.04 (0.51-2.09)	0.919
**Age (<65 vs. ≥65)**	1.40 (0.67-2.96)	0.372	0.90 (0.40-2.03)	0.797
**Gender (male vs. female)**	0.55 (0.30-1.01)	0.054	1.02 (0.51-2.05)	0.958
**BRAF status (mutant vs. WT)**	1.30 (0.68-2.48)	0.426	1.13 (0.54-2.37)	0.741
**Primary melanoma type (mucosal vs. cutaneous)**	1.28 (0.50-3.25)	0.606	1.16 (0.35-3.88)	0.804
**Pre-treatment LDH level** **(>ULN vs. normal)**	1.76 (0.95-3.24)	0.070	2.69 (1.31-5.50)	0.007*
**Brain metastases (yes vs. no)**	2.01 (1.09-3.72)	0.025*	1.32 (0.64-2.69)	0.453
**Liver metastases (yes vs. no)**	2.01 (1.04-3.88)	0.038*	1.65 (0.80-3.42)	0.177

I/N, ipilimumab/nivolumab; WT, wildtype; LDH, lactate dehydrogenase; ULN, upper limit of normal; CI, confidence interval.

^*^indicates statistical significance of p < 0.05.

^†^response assessment after 1 or 2 doses of I/N.

The multivariable Cox regression analysis (**Table 3**) found that PD (vs CBR) as initial response after 1 or 2 doses of I/N [HR: 8.04, 95% CI, 3.31-19.53; p<0.0001], treatment with 1 or 2 doses (vs 3 or 4 doses) of I/N [HR: 2.09, 95% CI, 1.04-4.19; p=0.038], and pre-treatment LDH levels greater than the upper limit of normal (vs normal) [HR: 2.69, 95% CI, 1.31-5.50; p=0.007] were associated with inferior OS.

**Figure 3:**
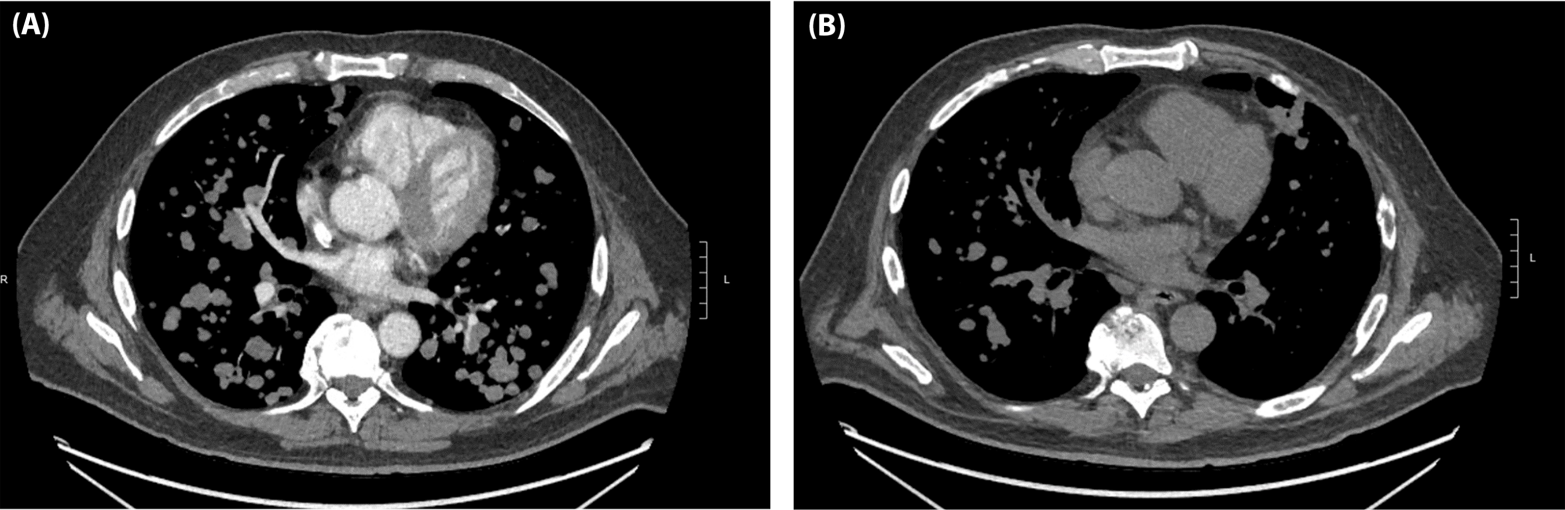
A 52-year-old man with BRAF-wildtype, metastatic melanoma with lung, liver, spleen, and brain involvement who received dose 1 of ipilimumab/nivolumab on 11/15/19 and dose 2 on 12/03/19. **(A)** Baseline CT chest on 11/06/19 and **(B)** CT chest on 12/17/19.

**Figure 4:**
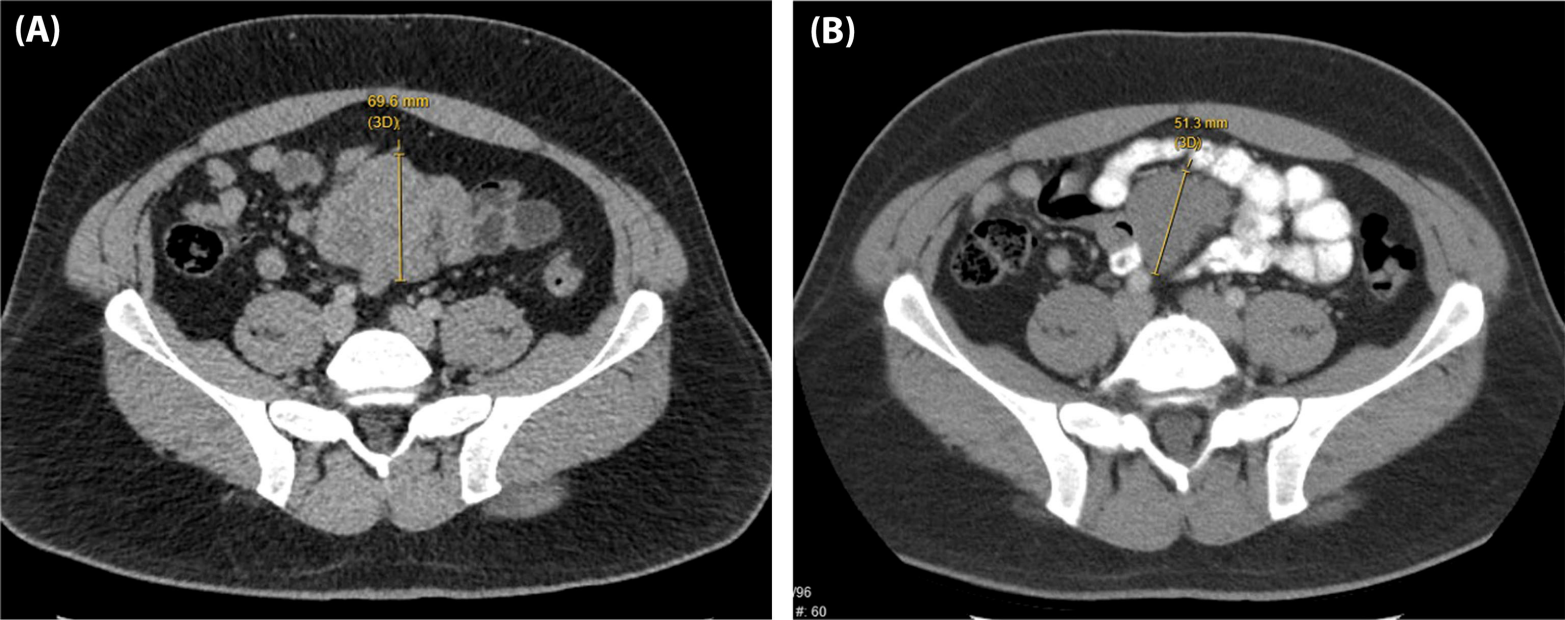
A 37-year-old man with BRAF-wildtype, metastatic melanoma with lung, bone, mesentery, and brain involvement who received dose 1 of ipilimumab/nivolumab on 06/23/17 and dose 2 on 07/15/17. **(A)** Baseline CT abdomen/pelvis on 05/23/17 and **(B)** CT abdomen/pelvis on 07/11/17.

Notable examples of patients who had early response assessment after 1 and 2 doses of I/N are shown in (**Figures 3**, **4**). The patient in (**Figure 3**) demonstrated lung metastases PR after 2 doses of I/N. The patient in (**Figure 4**) showed a mesentery metastasis PR after 1 dose of I/N.

Patients with CBR after 1 dose of I/N had improved PFS (HR: 0.16, 95% CI, 0.08-0.33; p<0.001) and OS (HR: 0.12, 95% CI, 0.05-0.32; p<0.001) compared to patients with PD (**Table 4**). Patients with CBR (vs PD) after 2 doses of I/N also had improved PFS (HR: 0.09, 95% CI, 0.05-0.16; p<0.001) and OS (HR: 0.07, 95% CI, 0.03-0.14; p<0.001). Significant correlation was found between initial response assessment and future best response after 1 dose of I/N (rho 0.5514; p<0.001) and after 2 doses of I/N (rho 0.6468; p<0.001) (**Supplementary Table 2**).

**Table 4:** Cox proportional regression model for progression-free survival and overall survival following initial response assessment (PD as the reference group).

Response assessment^†^		n	Progression-Free Survival		Overall Survival	
			Hazard Ratio (95% CI)	p-value	Hazard Ratio (95% CI)	p-value
**After 1 dose of I/N** ^‡^	PD	45	1.00		1.00	
	CBR (SD+PR+CR)	52	0.16 (0.08-0.33)	<0.001*	0.12 (0.05-0.32)	<0.001*
**After 2 doses of I/N** ^§^	PD	43	1.00		1.00	
	CBR (SD+PR+CR)	92	0.09 (0.05-0.16)	<0.001*	0.07 (0.03-0.14)	<0.001*

I/N, ipilimumab/nivolumab; CBR, clinical benefit response; PD, progressive disease; SD, stable disease; PR, partial response; CR, complete response; CI, confidence interval.

*indicates statistical significance of p < 0.05.

^†^Per RECIST v1.1 criteria.

^‡^Response assessed between 1st and 2nd dose of I/N. If 2nd dose was never received, then the response was assessed within 30 days after the 1st dose of I/N.

^§^Response assessed between 2nd and 3rd dose of I/N. If 3rd dose was never received, then the response was assessed within 30 days after the 2nd dose of I/N .

Among the cohort of patients with complete baseline characteristic and prognostic information and a CBR after the 1st or 2nd dose of I/N (n=94), univariate Cox regression (**Supplementary Table 3**) and multivariable Cox regression analyses (**Table 5**) were performed. The multivariable Cox regression analysis no longer demonstrated a significant difference in PFS [HR 0.95, 95% CI, 0.37-2.48; p=0.921] or OS [HR 1.04, 95% CI, 0.22-4.78; p=0.965] based on treatment with 1 or 2 (vs 3 or 4 doses) of I/N. The only variable that continued to show a significant difference in survival was presence of brain metastases [HR: 2.69, 95% CI, 1.08-6.66; p=0.033] for PFS.

**Table 5:** Multivariable Cox regression of progression-free survival and overall survival on the number of I/N (ipilimumab/nivolumab) doses and prognostic variables among patients who had clinical benefit response after 1 or 2 doses of I/N. n = 94.

	Progression-Free Survival		Overall Survival	
Variable	Hazard Ratio (95% CI)	p-value	Hazard Ratio (95% CI)	p-value
**I/N doses (1 or 2 vs. 3 or 4)**	0.95 (0.37-2.48)	0.921	1.04 (0.22-4.78)	0.965
**Time to initial assessment**	0.72 (0.26-1.94)	0.512	0.83 (0.19-3.57)	0.804
**Age (<65 vs. ≥65)**	0.70 (0.24-2.11)	0.531	0.61 (0.14-2.68)	0.515
**Gender (male vs. female)**	0.62 (0.24-1.63)	0.334	2.00 (0.44-9.09)	0.371
**BRAF status (mutant vs. WT)**	1.45 (0.56-3.75)	0.445	0.88 (0.22-3.42)	0.849
**Primary melanoma type (mucosal vs. cutaneous)**	2.22 (0.65-7.59)	0.206	2.38 (0.36-15.85)	0.371
**Pre-treatment LDH level (>ULN vs. normal)**	0.86 (0.34-2.16)	0.747	1.53 (0.37-6.21)	0.555
**Brain metastases (yes vs. no)**	2.69 (1.08-6.66)	0.033*	2.95 (0.86-10.14)	0.087
**Liver metastases (yes vs. no)**	2.00 (0.81-4.94)	0.133	1.55 (0.65-0.42)	0.510

I/N, ipilimumab/nivolumab; WT, wildtype; LDH, lactate dehydrogenase; ULN, upper limit of normal; CI, confidence interval.

^*^indicates statistical significance of p < 0.05.

We used a two-sample t-test in the PASS (Power Analysis and Sample Size) 2019 software to calculate the sample size needed to have 80% power of detecting the differences among two groups at 0.05 significance level. According to our output, we needed a total of 124 total patients to have an 80% chance of detecting a difference of five units among two groups. For a total of 94 total patients in our two-group comparison (**Table 5**), that difference will be within 0.5-0.8 with 80%. Thus, our sample size was powered enough to draw the conclusions of the study.

A multivariable Cox regression analysis of PFS and OS on the patients who had PD after 1 and/or 2 doses of I/N is displayed in (**Supplementary Table 4**). Although no difference in PFS and OS was seen after adjustment by I/N doses, it is difficult to draw this conclusion based on the small sample size (n=43).

## Discussion

In this retrospective analysis, patients with unresectable stage III or metastatic melanoma treated with standard combination I/N frequently receive less than 4 induction doses due to irAEs or progression. We found that a favorable early response, as measured by clinical benefit response (CBR) after 1 or 2 doses of I/N, may be a positive predictive marker for long-term survival in advanced stage melanoma. Patients who have CBR after 1 or 2 doses of I/N may have a similar survival benefit with fewer doses of I/N (1 or 2 vs 3 or 4 doses), though this requires further validation. As our study was non-interventional, treatment de-escalation was not offered based on an early favorable response. Patients who tolerated treatment were given I/N up to the planned 4 doses. Overall, our findings suggest that four I/N induction doses may not be necessary to have clinical benefit in a subset of patients.

Despite its demonstrated efficacy in advanced melanoma, combination I/N is associated with significant treatment-related toxicities. There is a high economic burden of ICI therapy, in part due to toxicity management (14). In our study, rates of any toxicities appeared similar regardless of the number of I/N doses received, but the percent of irAEs leading to discontinuation of I/N induction were 11% (21/199) following the 1st dose of I/N and 26% (51/199) following the 2nd I/N dose (**Table 2**). This raises the possibility that patients who would have been candidates for more combination doses could have received fewer doses in an effort to minimize irAEs. Rates of I/N discontinuation before 4 doses was higher in our cohort compared to historic data (3, 15), which may reflect varied patient populations and/or practice patterns. As noted by other authors, irAEs in a real-world population may be more expansive than those captured in clinical trials (15).

The distinctive biologic mechanism of immune-checkpoint blockade can also lead to unconventional tumor response patterns on standard imaging; a review of melanoma clinical trials found that pseudoprogression occurred in up to 10% of patients (16, 17). This has led to the creation of several ICI-adapted response criterion such as irRC (18), iRECIST (13), and irRECIST (19), but none have an explicit role in early disease assessment for ICI-treated patients. Despite the utilization of the conventional RECIST criteria (12), our study supports the notion that early progressors after the 1st or 2nd dose of I/N may predict worse long-term outcomes. As our comparator PD group encompassed would have included any patients with pseudoprogression, the negative prognosis associated with early-PD may be more dramatic in true progressors alone. One retrospective study found that pseudoprogression is associated with better outcomes than SD and true PD in ICI-treated NSCLC patients (20), but the comparison to those who achieve a PR or CR response remain uncertain.

To our knowledge, we are the first to assess the predictive role of interval response evaluation as early as after 1 dose of dual ICI therapy. By identifying those with a CBR early in treatment, we found that receiving fewer combination doses may not compromise long term survival. Although our study was observational, interventional studies evaluating toxicity rates in patients with early CBR electively receiving less than 4 I/N doses are warranted. Many clinical trial protocols have radiographic response evaluations no sooner than 9-12 weeks after ICI treatment (21–23). One small cohort study utilized FDG PET/CT imaging after 2 or 3 cycles of anti-PD-1 monotherapy to predict post-treatment progression in non-small cell lung cancer (NSCLC) patients (24). This response-adapted treatment strategy has been adopted in Hodgkin’s lymphoma and is being explored in multiple myeloma (25, 26). An ongoing clinical study in advanced melanoma is evaluating the efficacy of administering <4 induction doses contingent on a favorable anti-tumor effect after 2 doses of I/N (27). Utilizing a response-adapted therapy strategy may ultimately minimize unnecessary treatment, limit dose-dependent toxicities, and decrease healthcare expenses. Our study suggests early response evaluation during I/N induction could be utilized as a biomarker to one day impact future clinical practice decisions including: de-escalation of therapy in treatment-responders; escalation of therapy in partial-responders with ICI dose modification or addition of other anti-neoplastic agents; or salvage non-responders with other therapies.

Merits of our study include the incorporation of time to initial assessment in our Cox regression models to limit a guarantee-time bias. Our retrospective study was also inclusive of patients with CNS metastases to reflect real-world patients in clinical practice. There are several limitations to our study. All patients were treated with ipilimumab 3 mg/kg and nivolumab 1 mg/kg for induction, despite the available flipped dosing. As already discussed, rates of I/N discontinuation prior to the 4th dose were higher in our cohort compared to historic data (3, 15). Unlike the clinical trials that utilized RECIST v1.1 (2), our observed tripling of the median PFS is likely attributed to our use of iRECIST for defining progression. We also included a small proportion of patients who had prior therapy, acknowledging that previous studies have shown that patients exposed to I/N in the latter line setting have worse ORR and survival outcomes (28).

## Conclusion

Clinical benefit response (CBR) during induction with I/N may be predictive of long-term survival in advanced stage melanoma. Conclusions cannot be drawn at this time about a response-adapted treatment strategy or electively limiting I/N induction doses. Prospective clinical trials are required to validate these findings and to assess whether the elective administration of less than 4 doses of combination I/N in select patients better balances optimal treatment outcomes with diminished treatment related toxicity.

## Data Availability Statement

The raw data supporting the conclusions of this article will be made available by the corresponding author.

## Ethics Statement

The studies involving human participants were reviewed and approved by the University of Michigan institutional ethical guidelines and complies with the guidelines of the responsible governmental agency. Written informed consent for participation was not required for this study in accordance with the national legislation and the institutional requirements.

## Author Contributions

VM and CL were involved with the conceptualization, data acquisition, analysis/interpretation, methodology, and writing/revising of the original draft. AC and YS were involved in the analysis/interpretation and writing/revising. MS, JW, and GW were involved with the data acquisition and writing/revising of the original draft. LF and MG were involved in the methodology, writing/revising, and editing. All authors contributed to the article and approved the submitted version.

## Conflict of Interest

LF serves as a consulting or advisory role for Elsevier/*Via* Oncology and Hoosier Cancer Research Network. CL served as a consulting or advisory role for Bristol-Myers Squibb and Immunocore. LF receives research funding from Array BioPharma, Bristol-Myers Squibb, Incyte, Kartos Therapeutics, Merck, and Pfizer/EMD Serono. CL receives research funding from Bristol-Myers Squibb, Dynavax Technologies, and Genentech.

The remaining authors declare that the research was conducted in the absence of any commercial or financial relationships that could be construed as a potential conflict of interest.

## Publisher’s Note

All claims expressed in this article are solely those of the authors and do not necessarily represent those of their affiliated organizations, or those of the publisher, the editors and the reviewers. Any product that may be evaluated in this article, or claim that may be made by its manufacturer, is not guaranteed or endorsed by the publisher.
