# Supplementary material for: Early Response Assessment in Advanced Stage Melanoma Treated with Combination Ipilimumab/Nivolumab
Source: Front Immunol. 2022 Jul 6;13:860421. doi: 10.3389/fimmu.2022.860421 (PMC9296775; doi:10.3389/fimmu.2022.860421)
Supplement: Supplementary file 1 [file Table_1.docx]

**Supplementary Table 1.** Univariate Cox regression of progression-free survival and overall survival on the number of I/N (ipilimumab/nivolumab) doses and prognostic variables among patients who had response assessment after 1 or 2 doses of I/N.

|  | | **Progression-Free Survival** | | **Overall Survival** | |
| --- | --- | --- | --- | --- | --- |
| **Variable** | **n** | **Hazard Ratio (95% CI)** | **p-value** | **Hazard Ratio (95% CI)** | **p-value** |
| **PD vs CBR initial assessment**† | 139 | 7.00 (3.94-12.44) | <0.0001* | 8.39 (4.02-17.51) | <0.0001* |
| **I/N doses (1 or 2 vs 3 or 4)** | 139 | 1.41 (0.84-2.38) | 0.189 | 1.48 (0.81-2.72) | 0.207 |
| **Time to initial assessment** | 139 | 0.76 (0.46-1.28) | 0.305 | 0.85 (0.48-1.51) | 0.583 |
| **Age (<65 vs ≥65)** | 139 | 1.20 (0.66-2.18) | 0.546 | 0.86 (0.44-1.66) | 0.656 |
| **Gender (male vs female)** | 139 | 0.58 (0.35-0.96) | 0.033* | 0.78 (0.43-1.39) | 0.397 |
| **BRAF status (mutant vs WT)** | 139 | 1.14 (0.69-1.89) | 0.613 | 1.10 (0.61-1.97) | 0.759 |
| **Primary melanoma type (mucosal vs cutaneous)** | 122 | 1.42 (0.64-3.15) | 0.393 | 1.15 (0.41-3.22) | 0.797 |
| **Pre-treatment LDH level**  **(>ULN vs normal)** | 135 | 2.01 (1.21-3.34) | 0.007* | 2.69 (1.47-4.91) | 0.001* |
| **Brain metastases (yes vs no)** | 138 | 1.82 (1.10-3.03) | 0.021* | 1.62 (0.90-2.93) | 0.107 |
| **Liver metastases (yes vs no)** | 139 | 2.15 (1.30-3.56) | 0.003* | 2.40 (1.33-4.32) | 0.004* |

**Abbreviations:** I/N: ipilimumab/nivolumab; WT: wildtype; LDH: lactate dehydrogenase; ULN: upper limit of normal; CI: confidence interval

*indicates statistical significance of p<0.05

†response assessment after 1 or 2 doses of I/N
