# Supplementary material for: Early Response Assessment in Advanced Stage Melanoma Treated with Combination Ipilimumab/Nivolumab
Source: Front Immunol. 2022 Jul 6;13:860421. doi: 10.3389/fimmu.2022.860421 (PMC9296775; doi:10.3389/fimmu.2022.860421)
Supplement: Supplementary file 2 [file Table_2.docx]

**Supplementary Table 2.** Spearman’s rank correlation coefficient between initial response assessment and future best response.†

|  | **n** | **rho** | **p-value** |
| --- | --- | --- | --- |
| **Response assessment**‡ **after 1 dose of I/N** | 97 | 0.5514 | <0.001* |
| **Response assessment**‡ **after 2 doses of I/N** | 134 | 0.6468 | <0.001* |

**Abbreviations:** I/N: ipilimumab/nivolumab

*indicates statistical significance of p<0.05

†Per iRECIST criteria

‡Per RECIST v1.1 criteria
