# Supplementary material for: Early Response Assessment in Advanced Stage Melanoma Treated with Combination Ipilimumab/Nivolumab
Source: Front Immunol. 2022 Jul 6;13:860421. doi: 10.3389/fimmu.2022.860421 (PMC9296775; doi:10.3389/fimmu.2022.860421)
Supplement: Supplementary file 3 [file Table_3.docx]

**Supplementary Table 3.** Univariate Cox regression of progression-free survival and overall survival on the number of I/N (ipilimumab/nivolumab) doses and prognostic variables among patients who had clinical benefit response after 1 or 2 doses of I/N.

|  | | **Progression-Free Survival** | | **Overall Survival** | |
| --- | --- | --- | --- | --- | --- |
| **Variable** | **n** | **Hazard Ratio (95% CI)** | **p-value** | **Hazard Ratio (95% CI)** | **p-value** |
| **I/N doses (1 or 2 vs 3 or 4)** | 109 | 0.69 (0.33-1.43) | 0.321 | 0.70 (0.24-2.02) | 0.507 |
| **Time to initial assessment** | 109 | 0.82 (0.39-1.75) | 0.616 | 1.13 (0.42-3.00) | 0.812 |
| **Age (<65 vs ≥65)** | 109 | 1.06 (0.43-2.61) | 0.893 | 0.60 (0.19-1.93) | 0.392 |
| **Gender (male vs female)** | 109 | 0.88 (0.42-1.87) | 0.745 | 2.08 (0.58-7.48) | 0.260 |
| **BRAF status (mutant vs WT)** | 109 | 1.19 (0.57-2.49) | 0.634 | 0.79 (0.27-2.38) | 0.680 |
| **Primary melanoma type (mucosal vs cutaneous)** | 95 | 2.14 (0.72-6.37) | 0.172 | 2.16(0.46-10.01) | 0.327 |
| **Pre-treatment LDH level (>ULN vs normal)** | 108 | 1.13 (0.53-2.43) | 0.750 | 1.90 (0.65-5.60) | 0.243 |
| **Brain metastases (yes vs no)** | 108 | 1.96 (0.93-4.15) | 0.079 | 2.02 (0.70-5.86) | 0.194 |
| **Liver metastases (yes vs no)** | 109 | 1.78 (0.85-3.71) | 0.123 | 2.94 (1.02-8.51) | 0.047* |

**Abbreviations:** I/N: ipilimumab/nivolumab; WT: wildtype; LDH: lactate dehydrogenase; ULN: upper limit of normal; CI: confidence interval

*indicates statistical significance of p<0.05
