# Supplementary material for: Early Response Assessment in Advanced Stage Melanoma Treated with Combination Ipilimumab/Nivolumab
Source: Front Immunol. 2022 Jul 6;13:860421. doi: 10.3389/fimmu.2022.860421 (PMC9296775; doi:10.3389/fimmu.2022.860421)
Supplement: Supplementary file 4 [file Table_4.docx]

**Supplementary Table 4.** Multivariable Cox regression of progression-free survival and overall survival on the number of I/N (ipilimumab/nivolumab) doses and prognostic variables among patients who had PD after 1 and/or 2 doses of I/N. n=43

|  | **Progression-Free Survival** | | **Overall Survival** | |
| --- | --- | --- | --- | --- |
| **Variable** | **Hazard Ratio (95% CI)** | **p-value** | **Hazard Ratio (95% CI)** | **p-value** |
| **I/N doses (1 or 2 vs 3 or 4)** | 2.03 (0.88-4.67) | 0.095 | 1.57 (0.63-3.89) | 0.329 |
| **Time to initial assessment** | 0.36 (0.10-1.31) | 0.120 | 1.19 (0.51-2.81) | 0.686 |
| **Age (<65 vs ≥65)** | 1.40 (0.44-4.41) | 0.570 | 0.65 (0.21-2.02) | 0.458 |
| **Gender (male vs female)** | 0.33 (0.13-0.81) | 0.015* | 0.69 (0.27-1.75) | 0.438 |
| **BRAF status (mutant vs WT)** | 1.45 (0.62-3.41) | 0.391 | 1.51 (0.56-4.03) | 0.414 |
| **Primary melanoma type (mucosal vs cutaneous)** | 0.87 (0.23-3.22) | 0.835 | 1.99 (0.39-10.16) | 0.406 |
| **Pre-treatment LDH level (>ULN vs normal)** | 3.02 (1.09-8.40) | 0.034* | 3.26 (1.14-9.33) | 0.028* |
| **Brain metastases (yes vs no)** | 1.30 (0.55-3.04) | 0.547 | 0.84 (0.32-2.20) | 0.726 |
| **Liver metastases (yes vs no)** | 0.91 (0.32-2.55) | 0.858 | 0.90 (0.36-2.23) | 0.815 |

**Abbreviations:** I/N: ipilimumab/nivolumab; WT: wildtype; LDH: lactate dehydrogenase; ULN: upper limit of normal; CI: confidence interval

*indicates statistical significance of p<0.05
